# Prediction of cognitive performance in old age from spatial probability maps of white matter lesions

**DOI:** 10.18632/aging.102901

**Published:** 2020-03-19

**Authors:** Cui Zhao, Ying Liang, Ting Chen, Yihua Zhong, Xianglong Li, Jing Wei, Chunlin Li, Xu Zhang

**Affiliations:** 1 School of Biomedical Engineering, Capital Medical University, Beijing, China; 2 Beijing Key Laboratory of Fundamental Research on Biomechanics in Clinical Application, Capital Medical University, Beijing, China

**Keywords:** white matter lesions (WMLs), cognition, machine learning, aging

## Abstract

The purposes of this study were to explore the association between cognitive performance and white matter lesions (WMLs), and to investigate whether it is possible to predict cognitive impairment using spatial maps of WMLs. These WML maps were produced for 263 elders from the OASIS-3 dataset, and a relevance vector regression (RVR) model was applied to predict neuropsychological performance based on the maps. The association between the spatial distribution of WMLs and cognitive function was examined using diffusion tensor imaging data. WML burden significantly associated with increasing age (r=0.318, p<0.001) and cognitive decline. Eight of 15 neuropsychological measures could be accurately predicted, and the mini-mental state examination (MMSE) test achieved the highest predictive accuracy (CORR=0.28, p<0.003). WMLs located in bilateral tapetum, posterior corona radiata, and thalamic radiation contributed the most prediction power. Diffusion indexes in these regions associated significantly with cognitive performance (axial diffusivity>radial diffusivity>mean diffusivity>fractional anisotropy). These results show that the combination of the extent and location of WMLs exhibit great potential to serve as a generalizable marker of multidomain neurocognitive decline in the aging population. The results may also shed light on the mechanism underlying white matter changes during the progression of cognitive decline and aging.

## INTRODUCTION

White matter lesions (WMLs), also called white matter hyperintensities (WMH), refer to hyperintense signals on T2-weighted or fluid-attenuated inverse recovery (FLAIR) images. These are largely thought to be due to cerebral small vessel disease and are widely prevalent among elderly individuals [1]. Histopathologically, WMLs may reflect demyelination, axon loss, or gliosis of brain white matter [2, 3], and they are associated with an increased risk of vascular cognitive impairment and dementia [4]. Neuroimaging studies have provided strong evidence that WMLs may be a useful surrogate biomarker predictive of cognitive decline and progression to dementia [5]. Consistent with that idea, it has been estimated WMLs contribute to nearly half of dementias worldwide, though the mechanism remains unknown [6–8].

WMLs are often accompanied by impairments in executive function, processing speed, attention, and memory [9, 10], and the volume of WMLs is associated with cognitive decline in older adults independent of brain atrophy [11, 12]. Not surprisingly, resent work indicates that the cognitive impairment reflects not only the volume of WMLs but also their location. For example, declines in complex processing speed correlates mainly with anterior WML progression, while declines in visual-construction functions tend to correlate of posterior WML progression [13]. In addition, increased WML volume in the parietal lobes associates with an increased risk of incident dementia [14]. Still, the relationship between the anatomical location of WMLs and cognitive decline is poorly understood. Consequently, there is a need to better understand the mechanisms underlying the cognitive impairment associated with vascular risk factors and WMLs, and to improve diagnoses and interventions into vascular cognitive impairment and dementia in older subjects [15].

Over the last decades, rapid improvements in medical imaging and machine learning technology and greater availability of neuroimaging datasets have provided opportunities for automatic detection and early prediction of cognitive decline [16, 17]. In the present study, we investigated the association between spatial maps of WMLs and multidomain cognitive performance in elderly adults, using MRI (structural MRI and diffusion tensor imaging/DTI) and various neuropsychological assessments of cognition (nonimpaired, mild cognitive impairment/MCI and dementia) with participants in the Open Access Series of Imaging Studies-3 (OASIS-3) [18]. We also explored whether it is possible to predict individual differences in cognitive function using the spatial probability maps of WMLs.

## RESULTS

### Behavioral performance and WML burden

A total of 263 elderly subjects (aged 62-80 years), including 122 (46.39%) women, participated in this study. The demographic and neuropsychological features of the participants are summarized in Table 1. Among them, 207 subjects (78.71%) were cognitively normal; that is, their scores on the mini-mental state examination (MMSE) were within the normal range and their clinical dementia rating scores (CDRs) were equal to zero. The remaining 56 subjects (21.29%) were diagnosed with MCI or Alzheimer’s dementia. The volumes of white matter lesions extracted using the brain intensity abnormality classification algorithm (BIANCA) ranged from 1.21 ml to 41.63 ml (mean: 7.89 ml). Lesion maps of WMLs segmented by BIANCA overlapped well with the manually segmented lesion mask. Statistical results revealed that WML volume was significantly related to age (Pearson’s correlation coefficient r=0.318, p<0.001). Poor neuropsychological performance was associated with both age and lesion volume. With increasing age and WML volume, cognitive performance tended to decline. In addition, the correlation between cognition and WML volume was more significant than the correlation between cognition and age.

**Table 1:** Subject demographics and neuropsychological performance (from the OASIS3 dataset).

		**Subjects (N=263)**	**Correlation with age**	**Correlation with WMLs**
**Demographics**				
Age		72.78±4.23	-	0.318**
Gender (F/M)		122/141	-	-
Education		14.89±1.20	-	-
APOE ε4 status (n%)		109 (41.44)	-	-
WMLs volume (mL)		7.89±6.16	0.318**	-
Level of independence (n%)	Level 1	249 (94.68)	-	-
	Level 2	9 (3.42)	-	-
	Level 3	5 (1.90)	-	-
**Neuropsychological tests**				
CDR		0.10±0.37	-	-
MMSE		28.36±2.58	-0.062	-0.102
LOGIMEM		13.86±4.38	0.041	-0.195**
DIGIF		8.48±2.00	-0.035	-0.104
DIGIFLEN		6.70±1.11	-0.023	-0.074
DIGIB		6.55±2.25	-0.034	-0.107
DIGIBLEN		4.78±1.29	0.018	-0.083
MEMUNITS		12.69±4.88	0.022	-0.195**
MEMTIME		14.69±4.88	0.056**	0.033
ANIMALS		20.59±6.16	-0.175**	-0.192**
VEG		14.10±4.34	-0.138*	-0.168**
TRAILA		32.44±11.77	0.043	0.166**
TRAILB		88.03±49.69	0.124*	0.069
TRALIB-A		55.59±43.85	0.128*	0.034
WAIS		53.49±11.62	-0.129*	-0.285**
BOSTON		27.38±3.16	-0.075	-0.063

APOE= apolipoprotein E. Level of independence: 1 = Able to live independently, 2 = Requires some assistance with complex activities, 3 = Requires some assistance with basic activities. MMSE= mini-mental state examination; LOGIMEM=logical memory; DIGIF= digit span forward; DIGIFLEN= digit span forward length; DIGIFB= digit span backward; DIGIFBLEN= digit span backward length; VEG=vegetables; TRAILA=trail making A; TRAILB=trail making B; TRAIL B-A=TRAILB-TRAILA; WAIS= Wechsler Adult Intelligence Scale; BOSTON=Boston naming test. Pearson’s correlations, controlled for gender and education, were used to assess how cognitive performance related to age and volume of WMLs (White matter lesions). *p < 0.05, ** p < 0.01.

### Spatial maps of WMLs were predictive of cognitive performance

**Figure 1:**
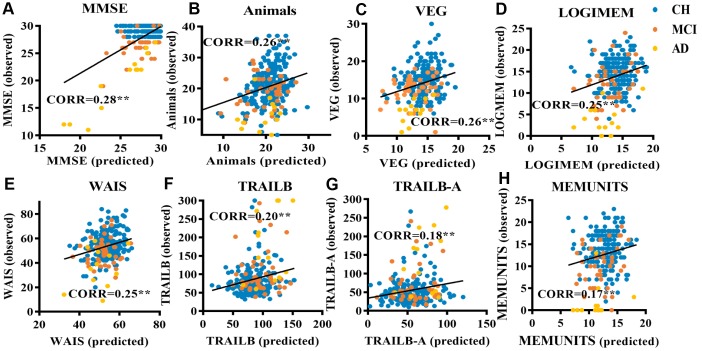
**Scatter plots relating cognitive performance predicted using a RVR model based on lesion probability maps of WMLs to observed performance in elderly individuals.** (**A**) RVR-MMSE; (**B**) RVR-ANIMALS; (**C**) RVR-VEG; (**D**) RVR-LOGIMEM; (**E**) RVR-WAIS; (**F**) RVR-TRAILB; (**G**) RVR-TRAIL B-A; (**H**) RVR-MEMUNITS. Scores of participants with cognitive impairment: participants with mild cognitive impairment (MCI) are colored orange, those clinically diagnosed with Alzheimer’s dementia (AD) are colored yellow. Cognitively healthy participants with WMLs are colored blue.

The results of predictions with voxel-level features derived from probability maps of WMLs are reported in Table 2. In 8 of the 15 neuropsychological testing scores, the predicted scores correlated highly with the actual scores (p<0.05). The predicted and actual scores from the MMSE showed the most significant correlations. The RVR model achieved a correlation coefficient (CORR) of 0.28 (p=0.003) and a normalized mean square error (norm MSE) of 0.38 (p=0.007). In addition, the predicted Category Fluency scores of ANIMALS and VEG also strongly correlated with the observed scores (ANIMALS: CORR=0.26, norm MSE=1.2, p<0.05; VEG: COOR=0.26, norm MSE= 0.61, p<0.05). The corresponding scatter plots illustrated in Figure 1 show the predicated clinical scores from the RVR model plotted against the observed scores.

**Table 2:** RVR model predictions of cognitive functions based on WMLs segmented using BIANCA.

**Neuropsychological tests**	**CORR**	**p**	**MSE**	**p**	**Norm MSE**	**p**
MMSE	0.28	0.003	8.05	0.007	0.38	0.007
ANIMALS	0.26	0.001	38.34	0.003	1.20	0.003
VEG	0.26	0.001	18.54	0.001	0.64	0.001
LOGIMEM	0.25	0.001	19.15	0.001	0.80	0.001
WAIS	0.25	0.001	135.42	0.006	1.81	0.006
TRAILB	0.20	0.002	2540.06	0.015	9.48	0.015
TRALIB-A	0.18	0.003	2016.58	0.030	7.52	0.030
MEMUNITS	0.17	0.003	24.94	0.040	1.08	0.040

### Weights in the RVR model and the association between diffusion metrics in the corresponding regions and cognition

**Figure 2:**
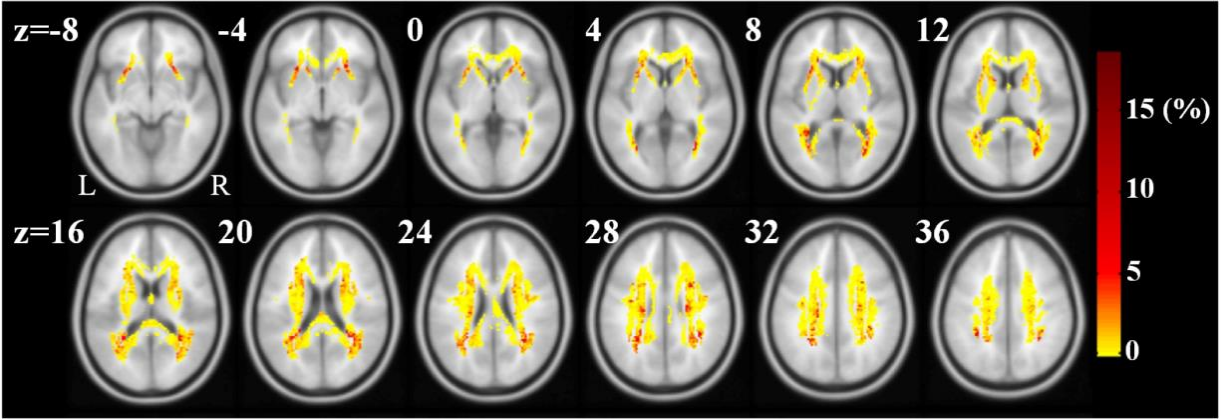
**Weight maps in the RVR-ANIMALS model.** Only voxels with positive weights and overlapping with JHU white-matter atlas are presented. The redder the color, the larger the weight of the voxel.

**Figure 3:**
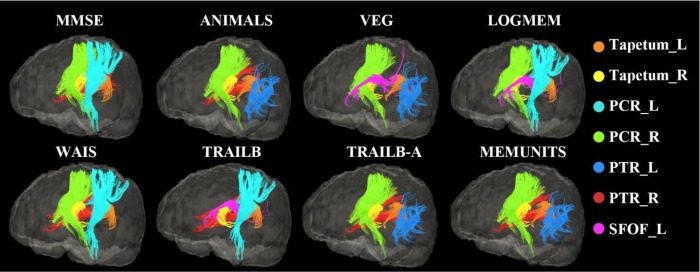
**White matter fiber tracts in which WMLs made a higher contribution to the prediction of cognitive performances than lesions located in other brain areas.** For each test, only the top 5 white matter tracts are displayed.

The top five regions contributing most to the RVR model for prediction of different measures of cognitive function are listed in Table 3 along with their weights, which are arranged from largest to smallest. The distributions of model weights were similar. White matter regions with large contributions to the RVR model mainly included the bilateral tapetum, posterior corona radiation, posterior thalamic radiation, and anterior limb of the internal capsule. Voxel-level weight maps of the RVR model predicting scores in ANIMALS is presented in Figure 2, where only voxels overlapping the JHU white-matter atlas are displayed. Overall, when compared to the anterior WMLs located in the frontal lobe, posterior WMLs located in the parietal and occipital lobes tended to have higher weights in the RVR model predicting cognition in the elderly subjects. The top five white matter regions with the highest weights in the prediction of cognitive performance are shown in Figure 3.

**Table 3:** Top five most relevant regions for prediction of cognitive performance based on the JHU white-matter atlas.

**Neuropsychological test**	**Hemisphere**	**Region description**	**Contribution (%)**	**ER**
MMSE	R	Tapetum	14.949	0.857
	L	Tapetum	10.453	1.714
	R	Posterior corona radiata	8.747	2.571
	R	Posterior thalamic radiation (include optic radiation)	7.837	4.143
	L	Posterior corona radiata	7.577	3.714
ANIMALS	R	Tapetum	13.042	0.857
	L	Tapetum	11.389	1.857
	R	Posterior corona radiata	9.290	2.429
	L	Posterior thalamic radiation (include optic radiation)	7.933	3.571
	R	Posterior thalamic radiation	6.366	4.429
VEG	R	Tapetum	19.474	0.857
	L	Tapetum	9.938	1.714
	L	Posterior thalamic radiation	7.496	3.714
	R	Posterior corona radiata	7.158	3.143
	L	Superior fronto-occipital fasciculus (could be a part of anterior internal capsule)	6.383	4.571
LOGIMEM	R	Tapetum	13.760	0.857
	L	Tapetum	10.637	1.714
	R	Posterior corona radiata	8.071	2.714
	L	Posterior corona radiata	7.703	3.286
	L	Superior fronto-occipital fasciculus	6.496	4.857
WAIS	R	Tapetum	13.155	1.000
	L	Tapetum	9.753	1.857
	R	Posterior corona radiata	7.332	3.143
	R	Posterior thalamic radiation	6.955	4.143
	L	Posterior corona radiata	6.493	4.000
TRAILB	R	Tapetum	14.602	0.857
	L	Tapetum	12.290	1.714
	L	Posterior corona radiata	7.631	2.857
	R	Posterior thalamic radiation	7.051	3.571
	L	Superior fronto-occipital fasciculus	6.096	4.571
TRAIL B-A	R	Tapetum	15.360	0.857
	L	Tapetum	12.307	1.714
	L	Posterior thalamic radiation	7.545	3.000
	R	Posterior thalamic radiation	7.252	3.286
	R	Posterior corona radiata	6.083	4.286
MEMUNITS	R	Tapetum	13.135	0.857
	L	Tapetum	10.157	1.714
	R	Posterior corona radiata	8.934	2.571
	L	Posterior thalamic radiation	7.518	3.714
	R	Posterior thalamic radiation	6.813	4.429

ER=Expected ranking. The region of the posterior thalamic radiation, including the optic radiation and the superior fronto-occipital fasciculus, could be part of the anterior internal capsule.

Table 4 summarizes the results of a multivariate linear regression analysis of the relation between eight cognitive test scores and the mean values of the diffusion metrics in the top five white matter regions with the maximum weights in the RVR model. For the cognitive test ANIMALS, four different types of diffusion indexes in the top five weight regions were all significantly related to the observed scores (fractional anisotropy /FA: F=4.760, p=0.001; mean diffusivity/MD: F=5.135, p=0.0003; axial diffusivity/AD: F=4.538, p=0.001; radial diffusivity/RD: F=5.049, p=0.0003). With the exception of the scores in the TRAIL B-A test, AD and RD correlated linearly with all the other seven items in the cognitive tests, which were predicted with the RVR model. The FA in the top five white matter regions was significantly related only to the scores of MMSE and ANIMALS tests.

**Table 4:** Results of multivariate linear regression relating cognitive performance and diffusion metrics.

**Cognitive tests**	**F**			
	**FA**	**MD**	**AD**	**RD**
MMSE	2.594*	4.106**	3.578**	4.275**
ANIMALS	4.760**	5.135**	4.538**	5.049**
VEG	1.280	5.572**	6.246**	5.069**
LOGMEM	0.444	2.925*	3.232**	2.770*
WAIS	2.273	2.904*	2.543*	2.962*
TRAILB	1.677	2.273	2.638*	2.931*
TRAIL B-A	1.929	1.833	1.630	1.938
MEMUNITS	0.587	1.929	3.824**	2.710*

## DISCUSSION

In the present study of 263 elderly individuals, we detected significant associations between increasing WML burden and declines across multiple cognitive functions. Using a multivariate modelling approach, we were able to predict multidomain cognitive performance in elders based on spatial probability maps of WMLs. We therefore suggest that variation in WMLs (extent and location) contributes to differences in cognitive dysfunctions in different domains.

Poor neuropsychological performance was significantly associated with larger WML volumes in elderly participants, which is generally in agreement with earlier studies [19]. Lesion volume correlated positively with increasing age and correlated negatively with cognitive function, especially language intelligence and memory. This association between WML burden and cognition was consistently found in both normally aging individuals and those with cognitive impairment, such as MCI and Alzheimer’s dementia [20–22]. Like medial temporal atrophy, apolipoprotein E (APOE) ε4 allele genotype, and β-amyloid burden, WML burden is a potentially useful surrogate biomarker with which to monitor cognitive performance and assess cognitive decline [23, 24]. Results from several studies support the hypothesis that this correlation between WMLs, especially periventricular lesions, and impaired cognition reflects a cholinergic deficiency [25–27].

A number of studies have focused on early prediction of cognitive impairment – i.e., the conversion from healthy cognition to MCI or from MCI to dementia. By combining large sample MRI data and machine learning models, several studies have achieved fairly high predictive accuracy [28–30]. Here, using a nonlinear multivariate regression model, RVR, we demonstrated that spatial probability maps of WMLs are predictive of multidomain cognitive performance by elderly individuals in tests of memory, language, intelligence, and executive functions (Table 2). Although recent evidence suggests the WML distribution may be a predictor of cognitive impairment, those results were controversial [31]. Some investigators were unable to detect an association between overall WML volume and a higher risk of dementia, but did find an association between lesions in specific brain regions (e.g., the parietal lobe) and the risk of dementia [14, 32, 33]. Indeed, in elderly individuals, WMLs were predictive of adverse cognitive outcomes reflecting changes in executive functions, memory, language and processing speed, as measured with the MMSE, trail making, Boston naming, and various other neuropsychological tests [34–36]. Our results with the RVR model are consistent with those earlier studies and further confirm that spatial maps of WMLs are predictive of many aspects of cognition in both healthy and cognitively impaired older people at the level of individuals.

Several specific regions, including the bilateral tapetum, posterior corona radiata, and posterior thalamic radiation (include optic radiation), showed strong associations with the prediction of cognitive performance, and these correlations were verified in DTI images. The tapetum is located on either side of the corpus callosum with fibers connecting the posterior corpus callosum and medial temporal lobe and covering the central part of the lateral ventricle. It has been reported that subjects with a family history of Alzheimer’s disease have a lower FA in the left tapetum [37], and that patients with Alzheimer’s disease have a lower FA and higher MD in these white matter regions [38, 39]. These regions were also associated with cognitive flexibility in young and middle-aged adults with dyskinetic cerebral palsy, a disease resulted from damage to the basal ganglia [40]. In addition, the bilateral posterior thalamic radiations, posterior corona radiata, and thalamocortical and corticocortical connections, which widely connect among the thalamus, parietal and occipital lobes, also appear to contribute greatly in the prediction of cognitive performance. This result is consistent with the significant association between thalamic pathology and memory loss in early Alzheimer’s disease, especially episodic memory, which is one of the earliest cognitive deficits in dementia [41, 42]. When predicting performance in neuropsychological tests, including the Wechsler Memory Scale-Revised, Category Fluency, and Trail Making Test (score: VEG, LOGIMEM and TRAILB), the superior fronto-occipital fasciculus, an association fiber tract connecting the frontal, occipital, parietal and temporal lobes, also exhibited high weight [43]. The superior fronto-occipital fasciculus is the only association fiber tract that projects medially to the thalamus and along the ventricle, and it is widely recognized to be an important connection between the insula and the parieto-frontal circuit, which are involved in crucial cerebral functions such as memory, language, emotion, and behavior [44].

In our results, WMLs located in posterior brain regions showed a slightly closer relation to cognitive impairment than other regions, which is consistent with several studies indicating that parietal and occipital lobes were the regions where WMLs preferentially occurred [45–49].

There are several limitations to the present study. A larger sample of participants will be needed verify the generalizability of our findings, especially the longitudinal data, which will be key to determining the predictive ability of WMLs for cognitive outcomes in elders. In addition, other modalities of neuroimaging, such as functional MRI (resting state and task driven) and positron emission tomography (PET), should also be examined. In future studies, combining WMLs with the other risk factors for cognitive decline in the elderly could further improve the predictive performance of our model and shed new light on the mechanism underlying WMLs in aging.

## CONCLUSIONS

In sum, both the volume and spatial distribution of WMLs are significantly associated with neuropsychological performance in elderly participants from a general population cohort. Multidomain cognitive performance could be predicted with the information on the intensity and spatial probability maps of WMLs. This may provide a basis from which to investigate the mechanisms underlying cognitive decline in aging, and help clinicians to identify elderly individuals at higher potential risk of early cognitive impairment.

## MATERIALS AND METHODS

### Participants

The participants in the current study are a cohort of elderly individuals from an ongoing project, known as the OASIS-3 study, which is an ongoing longitudinal neuroimaging, clinical, cognitive, and biomarker dataset for normal aging and Alzheimer’s Disease [18]. This dataset consists of >1000 participants aged 18-96, including cognitively normal adults and individuals at various stages of cognitive decline. A total of 263 subjects ranging in age from 55 to 80 years were included in the present study. These participants are from both genders and are all right-handed. Individuals with major psychiatric disorders or disease that could affect cognitive abilities were excluded. All participants completed a battery of neuropsychological test at the Alzheimer Disease Research Center (ADRC). These included the MMSE, Wechsler Memory Scale-Revised, Category Fluency, Boston Naming, Trail Making, Digit Span and Wechsler Adult Intelligence Scale-Revised [50]. A total of fifteen neuropsychological scores were included in this study. The participants’ clinical information, including education, APOE ε4 allele genotype, and level of independence were also included. Combined with the CDR scale and clinical dementia diagnoses collected in accordance with National Alzheimer’s Coordinating Center Uniform Data Set (UDS), 43 of the 263 available subjects had been diagnosed with MCI (CDR=0.5) and 13 were diagnosed with Alzheimer’s dementia (CDR>0.5) [51]. For each participant, T1 weighted (voxel size: 1.2×1.0547×1.0547 mm^3^) and T2 weighted FLAIR (voxel size: 0.8594×0.8594×5/6 mm^3^) were obtained. In addition, DTI images (voxel size: 2.5×2.5×2.5mm^3^, 65 directions, b0=1, b value=1000) were obtained from 124 of the subjects. MRI images were obtained using 3-T Siemens scanners.

### MRI imaging process

Identical imaging processing procedures were used for all subjects. The data was preprocessed using Statistical Parametric Mapping 12 (SPM12) (https://www.fil.ion. ucl.ac.uk/spm/software/spm12) running on MATLAB version 2016b, the PANDA toolbox (https://www.nitrc. org/projects/panda/), and the FMRIB software library v6.0 (https://fsl.fmrib.ox.ac.uk/fsl/fslwiki/FSL). Structural brain images as well as T1 and FLAIR images were stripped followed by bias field correction using FSL BET and FAST [52, 53]. FLAIR images were registered to the base modality T1 using linear-registration. The transformation between an individual’s native space and the standard Montreal Neurological Institute space coordinates was calculated as spatial features. After eddy current corrections, brain extraction, DTI index images, including FA, RD, AD, and MD, were calculated and normalized to the MNI standard space for further analysis.

### White matter lesions segmentation

We applied the segmentation algorithm BIANCA, a free FSL package, to automatically quantify the WMLs of our participants (https://fsl.fmrib.ox.ac.uk/fsl/fslwiki/BIANCA). BIANCA is a fully automated machine learning-based pipeline for detecting WMLs based on the k-nearest neighbor (KNN) classification algorithm, which offers highly flexible options for setting parameters such as modalities and location of training points [54]. In this study, we used T1 and FLAIR as features. T1 was set as the base space, and the training set consisted of 10 of the 263 subjects’ WML masks marked manually by an experienced neuroradiologist. Other options we used in BIANCA: spatial weighting=1; no patch; location of training points, any location for non-WMLs training points; number of training points, Fixed + unbalanced 2000 lesion points and 10000 non-lesion points. After segmentation, the probability maps of WMLs in T1 native space were extracted and volume of lesions was calculated. The intensity of each voxel was the probability that the voxel belongs to a WML and ranged from 0 to 1. Only voxels whose intensity exceeding 0.9 were retained; the others were set to zero. This threshold for the probability maps was set to obtain the best balance between false positive and false negative for the segmentation of WMLs, and was also suggested to be the optimal threshold for BIANCA [55]. Then lesion maps were spatially normalized to the standard space of 2×2×2 mm^3^. All registration steps were visually inspected.

### Prediction of cognitive performance using relevance vector regression

To investigate whether the spatial probability maps of WMLs were predictive of cognitive performance in elderly individuals, the relevance vector regression (RVR) model was applied. RVR is a sparse kernel method based on a probabilistic Bayesian framework with zero-mean Gaussian priors for the model weights, which are governed by hyperparameters [56, 57]. Specifically, the RVR model took the computed lesion maps of WMLs, excluding voxels locating in the cerebellum, as input vectors and the performances on a given neuropsychological test as targets. The posterior distributions of many of the model weights were sharply peaked at zero estimated with the training data, and the non-zero weights were “relevance vectors,” which were then used as the weighted relevance vectors to predict the target. The reliability of the WML-based predictive RVR model was measured using a 7-cross validation approach.

The RVR model provided a prediction of the clinical scores in a given test based on WML probability maps. The significance of the prediction performance was assessed using CORR, the MSE, and the norm MSE.

CORR provides a measure of the linear dependence between the targets and predictions; the higher the correlation, the more accurate the prediction. CORR was determined using the following formula:

$$\text{CORR}=\frac{\sum_{n}\left(y_{n}-\mu_{y}\right)\left(f\left(x_{n}\right)-\mu_{f}\right)}{\sqrt{\left\{\sum_{n}{\left(y_{n}-\mu_{y}\right)}^{2}\sum_{n}{\left(f\left(x_{n}\right)-\mu_{f}\right)}^{2}\right\}}}$$ (1)

MSE is a standard measure to assess goodness-of-fit for regression models, and different clinical scores have different scales. The higher the MSE, the less accurate are the predictions. MSE was calculated as:

$$\text{MSE}=\frac{1}{N}\sum_{n}{\left(y_{n}-f\left(x_{n}\right)\right)}^{2}$$ (2)

To minimize the effect of the scale of y on the MSE, we calculated the norm MSE:

$$\text{norm}\;\text{MSE}=\frac{MSE}{\left(y_{\max}-y_{\min}\right)}$$ (3)

*y_n_* and *f* (*x_n_*) denote the observed and estimated scores corresponding to the input predictors. *x_n_*, *μ_y_* and *μ_f_* are the sample means of *y_n_* and *f* (*x_n_*), respectively. N is the total number of subjects in the test sample. *y_max_* and *y_min_* are the maximum and minimum y, respectively.

### Association between diffusion indexes and the distribution of RVR weights

The model weights represent the contributions of each feature for the RVR predictive model. In this study, the region-level weight maps were respectively calculated based on the JHU white-matter label atlas containing 48 white matter regions in the brain and the weights of voxels in the same brain region averaged to display the decision functions of the predictive models [58]. These regions were ranked in ascending order based on their weights. The expected ranking (ER) of each region is the ranking averaged across folds. Additionally, to examine the association between cognitive functions and the weight distribution of the corresponding RVR prediction model, we first calculated the mean diffusion indexes in top five white matter regions with largest weights in the RVR model, including FA, AD, RD and MD. We then applied multivariate linear regression to assess how each type of diffusion measure in the five white matter regions was related to the corresponding scores in the cognitive performance tests.

### Statistics

**Figure 4:**
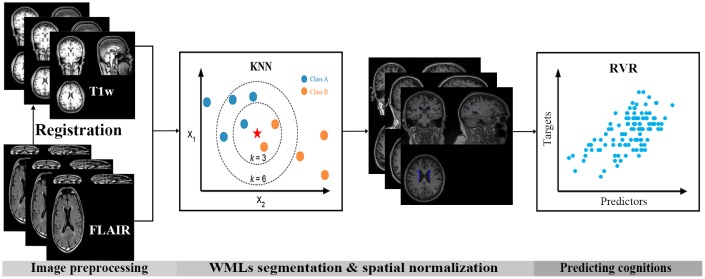
**Flow chart for analysis in the present study.** First, FLAIR images were registered to the corresponding individual’s T1 space. Then, the k-nearest neighbor (KNN) classification algorithm was used to segment the white matter lesions (WMLs) automatically. Finally, a machine learning model, relevance vector regression (RVR), was used to predict cognitive performance based on the spatial probability maps of the WMLs.

Partial Pearson’s correlations, controlled for gender and education, were used to assess how cognitive tests related to age and to the volume of WMLs detected by BIANCA. For the machine learning models used, permutation testing was performed to assess the models’ statistical significance. Specifically, each model was retrained 1000 times and P-values for CORR, MSE and norm MSE were calculated. Values of P < 0.05 were considered statistically significant. The overall procedure is shown in Figure 4.
