# Correction for: Prediction of cognitive performance in old age from spatial probability maps of white matter lesions

**DOI:** 10.18632/aging.203325

**Published:** 2021-07-14

**Authors:** Cui Zhao, Ying Liang, Ting Chen, Yihua Zhong, Xianglong Li, Jing Wei, Chunlin Li, Xu Zhang

**Affiliations:** 1 School of Biomedical Engineering, Capital Medical University, Beijing, China; 2 Beijing Key Laboratory of Fundamental Research on Biomechanics in Clinical Application, Capital Medical University, Beijing, China; 3 Equal contribution

**Keywords:** correction

Original article: Aging. 2020; 12:4822–4835.  . https://doi.org/10.18632/aging.102901

**This article has been corrected:** The authors requested to add Acknowledgments and replace Reference #18. The authors declare that these corrections do not change the results or conclusions of this work.

## Acknowledgments

Data were provided in part by OASIS-3: Principal Investigators: T. Benzinger, D. Marcus, J. Morris; NIH P50AG00561, P30NS09857781, P01AG026276, P01AG003991, R01AG043434, UL1TR000448, R01EB009352.

AV-45 doses were provided by Avid Radiopharmaceuticals, a wholly owned subsidiary of Eli Lilly.

## REFERENCES

18. LaMontagne PJ, Benzinger TLS, Morris JC, Keefe S, Hornbeck R, Xiong C, Grant E, Hassenstab J, Moulder K, Vlassenko A, Raichle ME, Cruchaga C, Marcus D. OASIS-3: longitudinal neuroimaging, clinical, and cognitive dataset for normal aging and Alzheimer disease. medRxiv. 2019: 12:13. https://doi.org/10.1101/2019.12.13.19014902

